# Supplementary material for: Towards a pragmatic use of statistics in ecology
Source: PeerJ. 2021 Sep 1;9:e12090. doi: 10.7717/peerj.12090 (PMC8418218; doi:10.7717/peerj.12090)
Supplement: Supplemental Information 1 — This shows some specifics on the relationship between NHT and IT approaches that are not detailed in the main text. [file peerj-09-12090-s001.pdf]

SUPPLEMENTARY ON LINE MATERIAL

S.1 Analytical relationship between NHT and IT approaches

Murtaugh (2014) and Aho et al. (2017) used the Log-likelihood ratio tests (LRT) to identify a mathematical relationship between p-values and Akaike weights. This demonstration is valid under the assumptions of LRT, namely that (i) the sample size is large enough to allow the distribution of deviances (minus twice likelihood ratios) under the null hypothesis to be approximated to a Chi-square distribution and (ii) the simpler model to be compared is a particular case of the more complex one (nested models). If these assumptions are met, the power of simple designs that can be translated into a single LRT is Aho et al. (2017)'s:

$$\beta = 1 - P_{\chi^2}(x, k = 1, \lambda = E^2) \tag{S-1}$$

where P_{χ^2} is the cumulative non-central distribution of chi-square values (x), with $k = 1$ degrees of freedom and noncentrality parameter λ equal to the square of effect size (E^2). For NHT, $x = 1.96^2$ and for IT $x = 2$. The left panel of Fig. S-1 shows the power of t-test predicted by equation S-1 for NHT and IT as a function of effect size for the t-test (equation 1 in the main text). As expected, the LRT approximation of t-test power matches the corresponding simulations. One can generalize this approximation for combinations of LRT tests. For instance, our linear regression simulation tested the effect of two putative independent variables (X_1 and X_2, see below), where only one of these variables affects the response Y. In this design, the LRT approximation for the power is:

$$\beta = [1 - P_{\chi^2}(x, k = 1, \lambda = E^2)] \times P_{\chi^2}(x, k = 1, \lambda = 0) \tag{S-2}$$

which is the product of the probabilities of rightfull conclusions for LRT tests for X_1 and X_2. The same rationale can be applied to any design that can be translated in a series of LRT's.

Edwards (1972) and Royall (2000) show many other instances of mathematical correspondence between inferences based on the Null Hypothesis Testing (NHT) and Information Theoretical (IT) approaches that do not rely on LRT. Here we show a simple example taken from Edwards (1972) to illustrate that the inferences done with both approaches tend to match as sample size increases, as found by Murtaugh (2014). Nevertheless, the convergence rates may differ, and thus for small to moderate sample sizes IT and NHT can lead to different inferences.

For many NHT standard tests, a correspondent "support test" can be defined as the degree of support the data provides for two alternative models (Edwards, 1972). The simplest case is a t-test for the null hypothesis that the sample comes from a Gaussian distribution with the mean fixed at a particular value. An alternative model is that the true mean of the distribution equals its maximum likelihood estimate (MLE), which is the sample mean. In both cases the standard deviation is set to its MLE, which is estimated from the sample. From an IT perspective the additional support that the alternative model has compared to the null model is the log-likelihood ratio. The minimum change in support required to reject the null model and choose the alternative model is a cutoff value of the log-likelihood ratio that can be expressed as function of t-statistic as:

$$\mid t_c \mid \; = \; \sqrt{(n-1)(e^{2L/n} - 1)} \tag{S-3}$$

where n is the sample size, L is the cutoff likelihood ratio and t_c is the critical t-value (Edwards, 1972). The right panel of Fig. S-1 shows the value of t_c calculated from equation S-3 and from the t-distribution as a function of sample size. Both tests statistics converge to the value of two as sample size increases, as expected with a Gaussian model. This convergence is slower for the t-test, which thus is more conservative in rejecting the null model under small sample sizes. The conclusions of the two tests are the same for sample sizes larger than 30, when the Gaussian distribution becomes a good approximation of the distribution of the t-statistics.

The geometry of the relationship between p-value and support

Figure S-2 shows the geometry behind the proof by Edwards (1972) that the p-value and log-likelihood ratios are monotonically related in the t-test, as in many other standard significance tests. The t-distribution

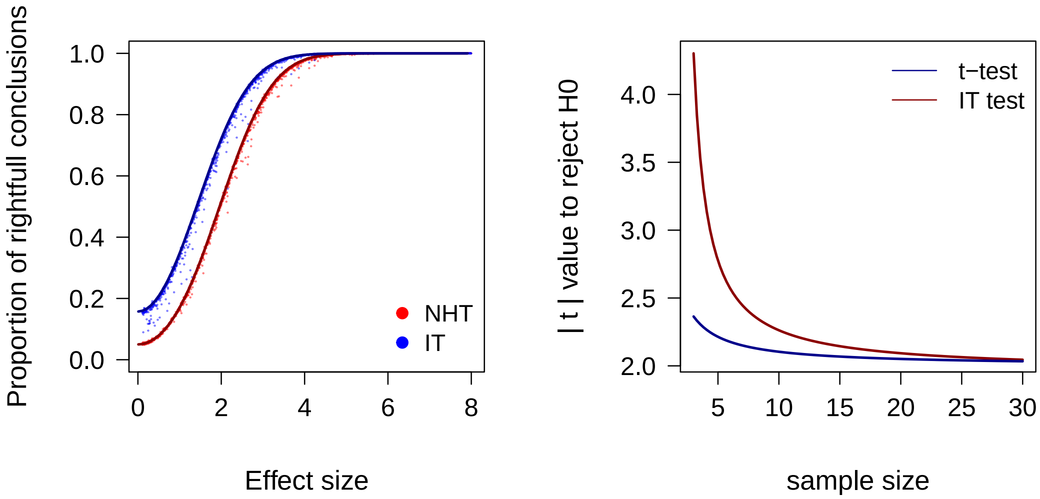

Figure S-1. Left panel: power (proportion of rightfull conclusions when the alternative hypotesis is true) in a t-test design, according to our simulations and the likelihood ratio test (LRT) approximation by Aho et al. (2017) for NHT (red) and IT (blue). Lines show the power predicted by the approximation and points are the proportions of simulations that ensued rightfull conclusions, as in Figure 1. **Right panel:** the critical value of the t-statistic and its IT correspondent as a function of sample size. In both cases the critical value t_c is the threshold value to reject the null hypothesis in a one-sample t-test design. The red line shows the critical value calculated from the t-distribution for a significance level of $\alpha = 0.05$. The blue line shows the critical value calculated from equation S-3 for a cutoff log-likelihood ratio of two. After Edwards (1972).

is a model for the t-statistic calculated from samples taken from the same Gaussian distribution. However, there are an infinite number of t-distributions for samples taken from Gaussian distributions that differ in some amount in their means. Among those alternatives will be the one that is best supported by the data. The lower the p-value of t under the null hypothesis, the higher the probability that the alternative, best supported t-distribution, assigns to this same value.

S.2 Adjusted IT criteria for uniformative models: effect on power, M-errors and S-errors

The t-test and correlation designs can be translated into two alternative models, which correspond to the null and alternative hypotheses in the NHT approach, as we detailed in the Methods section. Nevertheless, to translate ANOVA and linear regression designs to the IT approach we must fit more than one alternative model. For instance, in our linear regression example with two putative predictor variables, four additive models are possible:

- $E[Y] = a_0$
- $E[Y] = a_0 + a_1 X_1$
- $E[Y] = a_0 + a_2 X_2$
- $E[Y] = a_0 + a_1 X_1 + a_2 X_2$

The first model corresponds to the null hypothesis of no effect and the second model corresponds to the correct alternative hypothesis that only predictor X_1 has an effect on the expected value of the response variable ($E[Y]$). The fourth model also has the effect of X_2, which we set to zero in the simulated data. Random sampling variation will give this model an estimated value of the effect close to zero in each simulated fit. In this case the fourth model is equivalent to the correct model plus an uninformative parameter a_2. Nevertheless, the small estimated value of a_2 can sufficiently improve the fit to the observed data to make the AIC of this model with an uninformative parameter lower than the true model. The

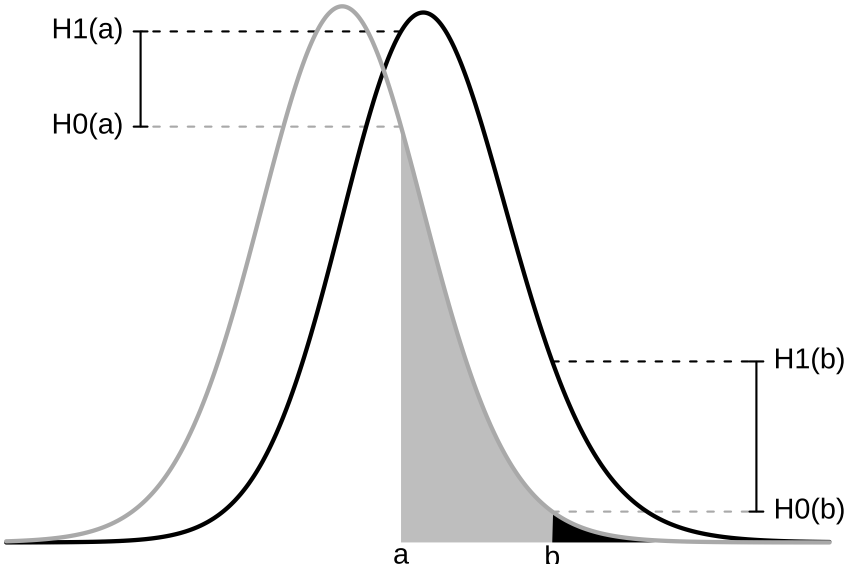

Figure S-2. The relationship between the p-value and the likelihood ratio in a t-test. The curve in grey is the standard t-distribution, which gives the probability density of a given t-value under the null hypothesis of no difference among population means. The curve in black is a non-central t-distribution, which gives the probability density of t-values under an alternative hypothesis that the population means differ to some amount. Points a and b are values of the t-statistic for two hypothetical testing situations. The areas below the curves are the p-values for $t = a$ (black + light grey area) and $t = b$ (only black area) under the null hypothesis. For each value of t, the likelihood ratio is the ratio between the probability density given by the two distributions ($\mathscr{L}_a = H_1(a)/H_0(a)$; $\mathscr{L}_b = H_1(b)/H_0(b)$). The comparison of situations a and b shows that the lower the p-value the higher the likelihood ratio H_1/H_0, and thus the stronger the support of H_1 over H_0. Akaike evidence weights w are proportional to likelihood ratios, and in such simple designs such as t-test w will increase monotonically as the p-value decreases. After Edwards (1972).

probability of this misleading selection converges to a value larger than zero as sample size increases (Geweke and Meese, 1981; Teräsvirta and Mellin, 1986), which means that AIC is not asymptotically consistent (although AIC is asymptotically efficient, see Aho et al., 2014, for a full discussion). This problem arises when the true model is surely among the competing ones and the purpose of model selection is to pick it (Aho et al., 2014), as is the case in simple significance test designs like those we simulated. To circumvent this problem we used the additional parsimony criterion proposed by Arnold (2010): to select the model with fewer parameters which was among the models with $\Delta AIC < 2$.

The figures below replicates the comparisons of power, S-errors and M-errors between NHT and IT approaches shown in figures 1 – 3 but also with the IT criterion without the parsimony correction described above. We also included the results for a simulation of the linear regression design with a correlation of 0.5 between the uninformative predictor (X_2) and the true predictor (X_1).

The power of the unadjusted IT criterion converges to the value 0.84 as effect size increases (Fig. S-3), because the probability of the selection of the model with an additional uninformative parameter converged to 0.16 in our simulations. This finding is in line with the theoretical upper bound of power

of the *AIC* model selection for two nested models (Teräsvirta and Mellin, 1986), as is the case for our simulations of ANOVA and linear regression. These results also support the proposition of Arnold (2010) that the power of *AIC* is bounded to $5/6$ when there is a model with a 'spurious variable'.

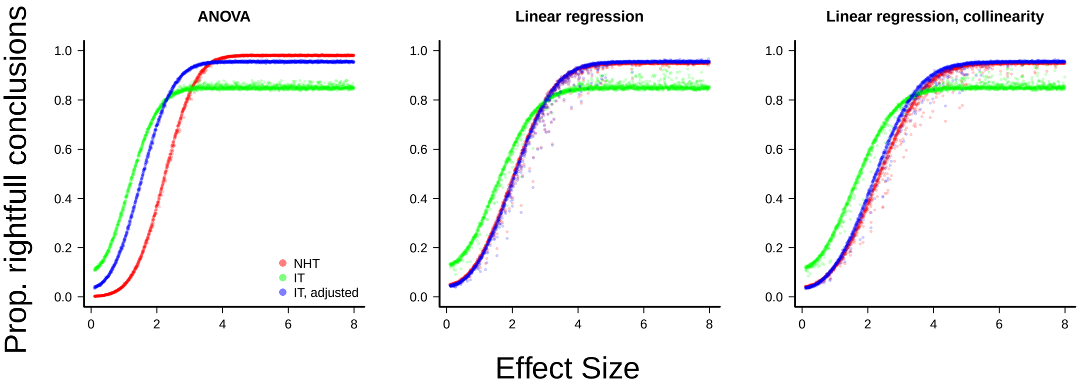

Figure S-3. The power of null hypothesis tests (NHT, red) and information-based model selection with and without the parsimony adjusting for uninformative parameters (green and blue, respectively. See Aho et al., 2014, and the text above), as a function of effect size, for ANOVA and linear regression designs. Each point is the proportion of the 10,000 simulations of a test instance from which the effect was detected. Each test instance used a different combination of effect size, standard deviation of the values, and sample size. Linear regression with collinearity was simulated with a correlation of 0.5 between the two predictor variables.

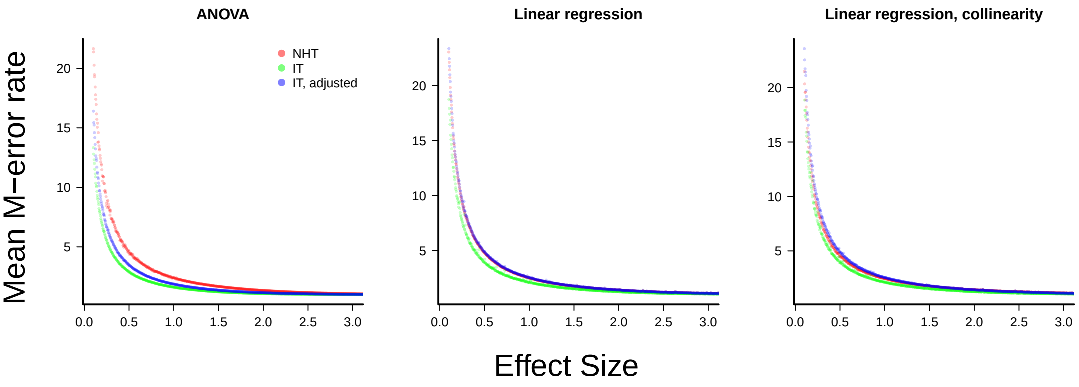

Figure S-4. The mean type-M error (exaggeration rate) of null hypothesis tests (NHT, red) and information-based model selection with and without the parsimony adjusting for uninformative parameters (IT, green and blue, see Aho et al., 2014, and the text above), as a function of effect size, for ANOVA and linear regression designs. Each point represents the simulations of a test instance from which an effect was detected. Each test instance used a different combination of effect size, standard deviation of the values and sample size and was simulated 10,000 times. The M-error is the absolute ratio between the effect size estimated and the true effect size (Gelman and Carlin, 2014), which was estimated from the mean of this ratio for each test instance. Linear regression with collinearity was simulated with a correlation of 0.5 between the two predictor variables.

S.3 R codes for the simulations

Functions in R to perform the simulations with any combination of parameters, the R scripts of the simulations, and the resulting simulated data used in this paper are available at `https://github.com/piklprado/NHTxIT`.

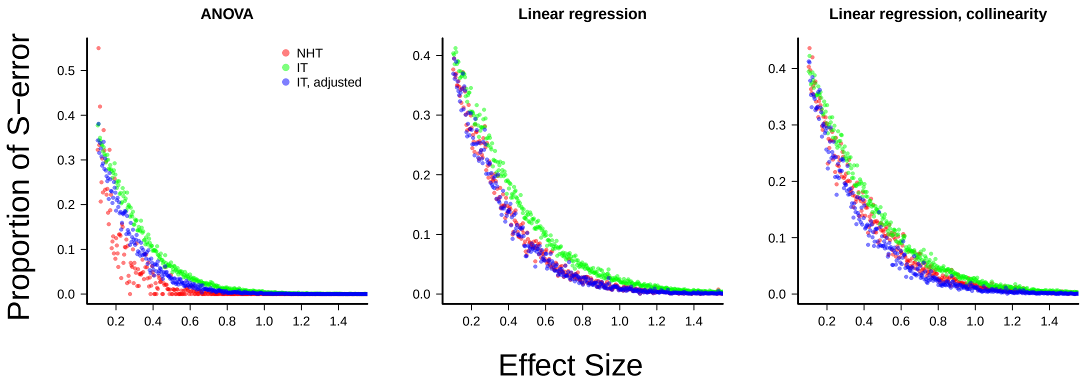

Figure S-5. The mean type-S error of null hypothesis tests (NHT, red) and information-based model selection with and without the parsimony adjusting for uniformative parameters (IT, green and blue, see Aho et al., 2014, and the text above), as a function of effect size, for ANOVA and linear regression designs. Each point represents the simulations of a test instance from which an effect was detected. Each test instance used a different combination of effect size, standard deviation of the values and sample size and was simulated 10,000 times. The S-error is the probability of detecting an effect of an opposite sign of the true effect (Gelman and Carlin, 2014). For each test instance we estimated S-errors from the proportion of simulations that detected an effect of the opposite sign. Linear regression with collinearity was simulated with a correlation of 0.5 between the two predictor variables.

REFERENCES

Aho, K., Derryberry, D., and Peterson, T. (2014). Model selection for ecologists: the worldviews of AIC and BIC. *Ecology*, 95(3):631–636

Aho, K., Derryberry, D., and Peterson, T. (2017). A graphical framework for model selection criteria and significance tests: refutation, confirmation and ecology. *Methods in Ecology and Evolution*, 8(1):47–56

Arnold, T. W. (2010). Uninformative parameters and model selection using akaike's information criterion. *The Journal of Wildlife Management*, 74(6):1175–1178

Edwards, A. W. F. (1972). *Likelihood: An Account of the Statistical Concept of Likelihood and its Application to Scientific Inference*. Cambridge University Press, Cambridge

Geweke, J. and Meese, R. (1981). Estimating regression models of finite but unknown order. *International Economic Review*, pages 55–70

Murtaugh, P. A. (2014). In defense of P values. *Ecology*, 95(3):611–617

Royall, R. (2000). *Statistical Evidence: A Likelihood Paradigm*. Chapman & Hall, London

Teräsvirta, T. and Mellin, I. (1986). Model selection criteria and model selection tests in regression models. *Scandinavian Journal of Statistics*, pages 159–171
